# Saving ambulance resources: a service evaluation of the identification of non-viable out-of-hospital cardiac arrest in London by advanced paramedic practitioners in critical care

**DOI:** 10.29045/14784726.2024.3.8.4.38

**Published:** 2024-03-01

**Authors:** Nick Brown, Chelsey Pike

**Affiliations:** London Ambulance Service NHS Trust ORCID iD: https://orcid.org/0000-0002-7257-536X; London Ambulance Service NHS Trust

**Keywords:** advanced paramedic, ambulance, cardiac arrest, savings, targets

## Abstract

**Background:**

Advanced paramedic practitioners in critical care (APPCCs) are advanced clinical practitioners focused on the delivery of pre-hospital critical care. While working in an ambulance control room setting, APPCCs seek to identify emergency calls appropriate for operational APPCCs to attend. These would include out-of-hospital cardiac arrest (OHCA). Through interrogation of incoming emergency calls they are also able to identify OHCA calls where resuscitation may be futile. In these cases, and within a governance framework, they stand down multiple ambulance responders, leaving only the closest responding resource to attend, thereby ‘saving resources’ that can be re-directed to other waiting emergency calls. It is believed that this is the first initiative of this nature in the United Kingdom.

**Methods:**

A three-year retrospective service evaluation of data was undertaken. The aim was to quantify the number of ‘saved resources’, including both double crewed ambulances (DCAs) and solo (single-person) responders, and furthermore to equate those savings into potential hours saved, using average known job cycle times (JCTs). Additionally, safety was assessed by searching all mandated incident reports for occasions where, despite cancellation of resources by an APPCC, resuscitation was commenced by the first response to scene.

**Results:**

A total of 13,356 ambulance resources were saved. Of these, 6593 (49.4%) were DCAs and 6763 (50.6%) were solo responders. Using the average JCT for deceased patients of 104.8 minutes, the total time saving equated to 23,328.48 hours of work or 1944.04 12-hour shifts. When considering DCAs alone, the average JCT for obviously deceased patients was 110.9 minutes. This equates to 12,186.1 hours of work or 1015.5 12-hour shifts. A total of 15 incident reports were identified. All had been investigated, revealing appropriate decision making in cancelling ambulance resources. No patient harm was identified.

**Conclusion:**

APPCCs working within a governance framework safely saved a significant number of ambulance resources over a three-year period. Perceived benefits include ‘freeing up’ DCA and solo responders, allowing them to be redirected to other emergency calls, leading to potential improvement in response times for patients waiting for an ambulance resource.

## Introduction

The incidence of out-of-hospital cardiac arrests (OHCAs) in the United Kingdom is approximately 55 per 100,000. There are over 30,000 OHCAs per year where emergency medical services (EMS) attempt resuscitation (Perkins et al., 2021). The London Ambulance Service NHS Trust (LAS) is the single EMS provider for the Greater London regions, serving a population of 8.2 million distributed throughout an area of 1579 km^2^. Between April 2021 and March 2022, the LAS attended 12,239 patients who had suffered a cardiac arrest. However, resuscitation was only attempted in 4366 (35.7%) patients. A total of 4984 were found either to be obviously deceased, making a resuscitation attempt futile, or to have a valid ‘do not attempt cardiopulmonary resuscitation’ (DNACPR) or equivalent in place (n = 2849). Additionally, 40 patients (0.3%) had received a shock from a public access defibrillator and were no longer in cardiac arrest (London Ambulance Service NHS Trust, 2022).

Within the LAS, the vast majority of OHCA patients are identified during the 999 call-handling process using the advanced medical priority dispatch system (AMPDS). Emergency call handlers (ECHs) answering 999 calls in the ambulance control room gather information pertaining to the location of the incident and the presenting condition of the patient, and deliver instruction to bystanders in how to perform cardiopulmonary resuscitation (CPR). Ambulance resources are simultaneously dispatched during this process. OHCA calls to ambulance services are triaged as Category 1 (highest priority), with a national ambulance response target time of seven minutes (or 15 minutes at the 90th centile) (NHS England, 2018). Within London, a minimum of four clinicians are dispatched to OHCA calls and paramedic attendance is mandated. Ambulance staff who attend OHCA calls can include emergency medical technicians, paramedics, clinical team managers, incident response officers and advanced paramedic practitioners. Although AMPDS has improved identification of non-breathing patients (Heward et al., 2004) from a call-taking perspective, it is not always possible to ascertain whether the patient is obviously deceased or whether CPR is appropriate. This could be due to the emotional state of the caller, language difficulties or a lack of circumstantial knowledge. Unless there is an unequivocal reason not to do so, CPR is usually instructed by the ECH.

Solo responding advanced paramedic practitioners in critical care (APPCCs) working for the LAS augment the standard pre-hospital response to high-acuity patient groups such as cardiac arrest, medical emergencies and major trauma. APPCCs have postgraduate education and training and experience in critical care. At any one time, a maximum of five APPCCs are available across London. The APPCC operating model requires all APPCCs to rotate through the ambulance control room, where their primary function is to identify appropriate incidents to task operational APPCCs to.

Enhanced interrogation of emergency calls most commonly involves the APPCC silently listening in to incoming calls to gain greater insight into the information being conveyed by the caller. ECHs are not clinicians, and follow an algorithmic approach to gather information and to support and advise the caller. This system facilitates a low threshold for the initiation of CPR for patients identified as not breathing. Conversely, APPCCs are advanced clinical practitioners who operate autonomously with guidelines. Using clinical acumen, APPCCs can be in a better position to contextualise information being relayed by a caller and thereby pick up on signals that might suggest resuscitation is not appropriate.

There is an obvious ambulance resource burden for active OHCA management. However, multiple ambulance crews arriving at a patient who is obviously deceased also spend time on scene investigating events, supporting families emotionally and practically, documenting events and handing over to police in cases of unexpected death. Data show that the average job cycle time (JCT) for double-crewed ambulances (DCAs) and solo (single-person) ambulance responders attending an obviously deceased patient is 104.8 minutes (London Ambulance Service NHS Trust, 2023). JCT refers to the total time that an ambulance resource is engaged in an emergency call and unavailable for other work.

In 2017, formal guidance was introduced allowing APPCCs to cancel resources from OHCA calls where reasonable information was provided for a patient to be considered obviously deceased, or where resuscitation would be futile. It is believed this was the first initiative of this nature in the United Kingdom. Decision making in these circumstances is multi-factorial and requires context, but examples of information that might contribute to such a decision include:

- mention of the patient being cold and stiff in a warm environment;
- a non-witnessed collapse;
- the patient not seen alive for several hours;
- a patient found by a carer or family member during a morning visit;
- evidence of decomposition or putrefaction;
- injuries incompatible with life;
- a DNACPR directive detailing a patient’s wish not to be resuscitated; or
- evidence of end-of-life care.

In these scenarios, the closest ambulance resource is still kept ‘running’ to the call under standard emergency ‘blue-light’ conditions, to provide CPR and defibrillation if deemed necessary. These are the two activities for which there is the most evidence of benefit in OHCA (Soar et al., 2021). If resuscitation is deemed appropriate, further ambulance resources are reinstated following a medical report to the control room. Cancelling unnecessary emergency resources enables further tasking of those ‘saved resources’ to other waiting emergency calls, while leaving a single resource to formally recognise death, support family or friends, complete documentation and hand over to the police or make contact with and report to a doctor.

A robust governance process is in place, utilising an incident reporting system. The APPCC and/or control room staff are required to complete an incident report when basic life support (BLS) is commenced and ambulance resources reinstated – contrary to the APPCC’s initial decision making. Each incident is then investigated by a senior member of the APPCC leadership team, examining call-taking records and patient care documentation for the appropriateness of the decision making and any patient harm caused. Although monthly reports detail the numbers of resources saved, no formal published longitudinal evaluation has been conducted examining the ambulance resource time saving and additionally the patient safety impact.

## Methods

This service evaluation involved retrospective observation aimed at amalgamating monthly data over a convenience sample of three years from June 2019, to ascertain ambulance hours saved and ‘put back into the system’. Average known JCT for obviously deceased patients was used to calculate the time saving related to DCA and solo ambulance responders. By way of comparison, LAS business intelligence data were used to ascertain the total number of patients attended where ambulance responders had recorded an ‘obviously deceased’ code for the same time period. Furthermore, patient safety was explored through the interrogation of completed incident reports and their findings.

For each day, on-duty control room APPCCs dynamically entered saved resources data onto a Microsoft Excel spreadsheet. The data comprised line entries that detail the following: call reference number; number of DCAs saved; numbers of solo responders saved; and brief clinical outcome (free text). At the end of each month, the numerical data were combined to produce a monthly total of DCA and solo resources saved, contained in another Microsoft Excel file. These historical records were amalgamated utilising Microsoft Excel to produce comparative numerical monthly data from June 2019 to July 2022. All calculations involving total additions, percentages, mean average and standard deviation (SD) were undertaken within Microsoft Excel.

Incident reports were searched from June 2019 to July 2022 for all entries that related to saved resources. Data gathered for each incident report included:

- whether the closest resource continued to scene;
- whether the Category 1 response time target was met;
- whether full advanced life support (ALS) was undertaken;
- patient outcome; and
- evidence of harm.

## Results

Over the three-year study period, there were 21,811 patients for whom an ambulance responder had recorded an ‘obviously deceased’ code. Over the same time period, a total of 13,356 individual ambulance resources were saved. Of these, 6593 (49.4%) were DCAs and 6763 (50.6%) were solo responders. The monthly mean average of resources saved was 371 (SD = 120.7). The monthly DCA mean average was 183.1 (SD = 74.1), and 187.7 (SD = 51.7) for solo responders.

Over the study period, the mean average JCT for a DCA and solo responder attending an obviously deceased patient was 104.8 minutes. Therefore, the total time saving equates to 23,328.48 hours of work or 1944.04 12-hour shifts, with a yearly mean average of 648.01 12-hour shifts. When considering DCAs alone, the mean average JCT for obviously deceased patients was 110.9 minutes. This equates to 12,186.1 hours of work or 1015.5 12-hour shifts.

**Figure 1:**
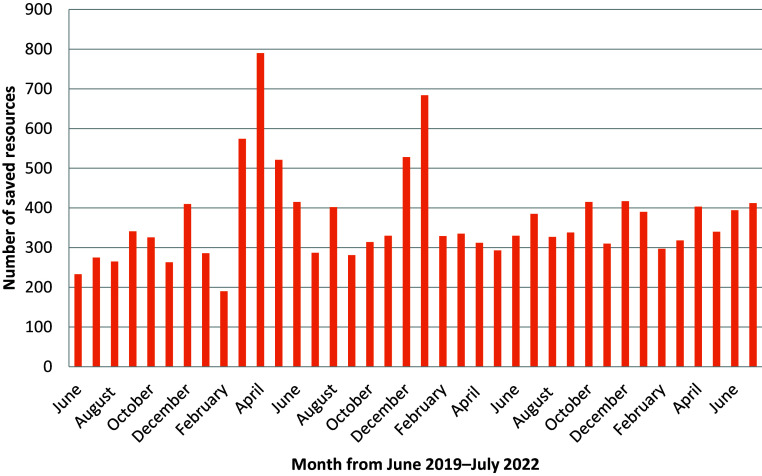
Figure 1. Resources saved from June 2019 to July 2022.

Over the three-year period, there were two notable peaks in saved resources data (Figure 1). The first was in the spring of 2020 and the second was in the winter of 2020. A lower average and slightly broader distribution was observed for the duration prior to the first peak in March 2020 (mean average of 285.8 saved resources, SD = 65.1), than that which followed the second peak ending February 2021 (mean average of 349 saved resources, SD = 42).

Fifteen incident reports were completed within the three-year period (Table 1). In all cases the closest dispatched resource continued to the call uninterrupted and in 12 (80%) instances arrived within the national standard for Category 1 response of seven minutes. Following an initial scene report, further resources were dispatched to assist and ALS was established in 10 cases. On three occasions, despite initial BLS, ALS was not established due to the identification of signs unequivocally associated with death. On a further two occasions the patient was not in cardiac arrest.

**Table 1.** Incidents reported following the initial request to reinstate ambulance resources.

Category 1 performance met	ALS established	Died on scene	Conveyed to ED	‘Harm’	Total
12	10	12	3	0	15

ALS: advanced life support; ED: emergency department.

Twelve of the 15 patients (80%) were declared deceased on scene. Two conveyed patients were not in cardiac arrest and a further elderly conveyed patient had extensive co-morbidities, and was palliated in the emergency department.

Following clinical investigation of the circumstances, all incident reports had been closed following a conclusion that decisions to cancel resources were reasonable and there was no evidence that cancellation decisions caused any patient harm.

## Discussion

Although response time improvements were seen in January 2023 (NHS England, 2023), in the United Kingdom demand on ambulance services has risen at an alarming rate. Figures for 2021/2022 show that for all triaged categories of calls, response times had increased. On average, the highest priority calls wait in excess of a minute and a half longer than the target of seven minutes, with the second highest priority calls waiting over double the target response time of 18 minutes on average (Alarilla et al., 2022). Ambulance service capacity is multi-factorial and includes workforce levels, integrity of fleet and equipment, the number of incidents requiring an ambulance response and JCT. The Association of Ambulance Chief Executives (2022) estimates that in November 2022 the total time lost to hospital handover delays alone (exceeding the 15-minute standard) was equivalent to 135,000 ambulance job cycles, or 23% of potential capacity.

Solutions to improving capacity will likely come from a combination of increased investment and alternative ways of working. Over the three years examined, a period equivalent to 12,186.1 hours of DCA work was saved (7450.57 hours for solo responders). This was additional time that allowed ambulance resources to be available for tasking to other emergency calls.

JCT mean average for all calls within the LAS was 104.4 minutes across all responder groups in 2022 (London Ambulance Service NHS Trust, 2023). Solo responders and DCA JCT mean average were 67.3 minutes and 110.8 minutes, respectively. Using these figures, it is reasonable to approximate that the increase in capacity would equate to 6598.97 additional patients responded to by DCAs, with a further 6642.41 additional solo ambulance responses for the timeframe of the study.

Relating time savings to cost saving is not straightforward. In many organisations, certain costings apply regardless of productivity. Additionally, certain incidents requiring an ambulance response draw on more resources than others. Nevertheless, ambulance patient-level activity and associated costing has been pursued. NHS data from 2019 to 2020 highlight ambulance costs of £2.3 billion over 10.7 million incidents, averaging £214.95 per incident (NHS Digital, 2021). Therefore, the study figure of 6598.97 DCA attendances saved might broadly compare to a cost saving of £1,418,470.19, following this methodology.

Two peaks were observed in the data in the spring of 2020 and over the following winter. These periods align with the two significant spikes in COVID-19 cases seen in London, and the associated rise in excess death (Office for National Statistics, 2023). During some of these ‘COVID months’, APPCC staffing was occasionally increased within the control room. In addition to the rise in 999 calls for OHCA patients, there would have been extra capacity to identify calls related to obviously deceased patients and to act to save multiple responses, particularly at a time when the ambulance service was overwhelmed with demand. The overall raised level of saved resources activity post COVID-19 may relate to a continued focus by APPCCs on saving resources following that period of excess death.

There are potentially additional benefits to redirecting resources to other incidents and improving response time for other waiting emergencies. Most OHCAs occur in the home environment (OHCAO Project Team, 2018) and will therefore likely involve an emergency call from family members. They will naturally expect a timely response from the ambulance service. However, in cases where someone is obviously deceased, there is also an opportunity to facilitate dignity in death. In these situations, the arrival of several emergency vehicles and subsequent entry of multiple uniformed clinicians plus their equipment may be overwhelming, stressful and add an unnecessary burden for grieving relatives. By contrast, the arrival of a single resource to assess the patient, confirm death, break bad news and offer support going forward may be a calmer, more considered and preferable experience.

Any systems should try to avoid bias. It might be suggested that advising the closest ambulance responder that their patient may be obviously deceased could engender a confirmation bias (NHS England National School of Healthcare Science, 2022) towards recognising a death rather than attempting resuscitation. It is worth highlighting that within the message sent to responding resources, ambulance staff are advised to call immediately if resuscitation is initiated. Additionally, clinicians have clear guidance on the recognition of life extinct (ROLE) criteria (Joint Royal Colleges Ambulance Liaison Committee & Association of Ambulance Chief Executives, 2023). Furthermore, electronic patient care records (ePCRs) are completed for each case attended, and clinical team managers are involved in the audit of a percentage of attended cases and their associated ePCRs. In this way, governance around decision making is more assured.

Conversely, there were a small number of occasions where resuscitation was initiated despite the control room APPCC determining that resuscitation was likely not appropriate. In any OHCA attendance, it is not uncommon for initial responders to establish BLS if the decision around whether to perform ROLE is equivocal. This allows clinicians to undertake those actions in a resuscitation attempt known to be most evidence based, namely chest compressions and the application of an automated external defibrillator (AED) (Soar et al., 2021). It could be said that these actions facilitate a clinical ‘holding pattern’ whereby more relevant facts can be established to assist decision making, such as time frames, co-morbidities, family wishes and clinical features. On at least three occasions identified by an incident report, subsequent findings revealed that further resuscitation was not in the patient’s best interest.

Front-line ambulance work carries a degree of psychological stress and moral burden (Miller, 2021). Dealing with death can be particularly psychologically impactful (Myall et al., 2020). In the process of cancelling down some of the ambulance responders, those individual staff members were not then subject to dealing with a deceased person and associated distraught family members. Indeed, it might be more challenging to find the right words to say to loved ones concerning a death than it would be to enact the largely algorithmic approach of resuscitation. Conversely, situations do arise whereby a solo responder attends a deceased patient after ‘back-up’ is cancelled down, leaving them to deal with this incident alone. Although not explicitly covered in the policy, control room staff and APPCCs are cognisant of this fact, and follow-up welfare calls can be made. In addition, all front-line crews have a number of options for on-scene or remote support and advice, should this be required. Furthermore, within the LAS, solo responders are among the more experienced front-line staff members, having undertaken prior DCA work, and are more likely aware of the demands of solo working. However, without continued focus on strategies to improve ambulance waiting times, the burden of solo responding without timely back-up could increase.

### Limitations

Limitations are conspicuous by the retrospective nature of the study, relying on APPCCs to self-report resources saved. Under-reporting is conceivable due to the busy nature of the control room setting and other activity that takes priority over this administrative function. Incident reporting compliance was not checked, and the investigation of incidents was carried out by managers within the APPCC programme. The study period ran over the COVID-19 pandemic, a time of increased death, which likely impacted on decisions to cancel resources.

## Conclusion

APPCCs operating within the ambulance control room are able to identify emergency calls made for an OHCA and where there is sound reason to believe resuscitation is futile. Within an agreed policy and governance framework they can safely stand down excess ambulance resources, while leaving the closest attending responder driving to scene to evaluate the clinical situation and provide a report. In doing so, within a three-year period 13,356 ambulance resources were saved, allowing them to be redirected to other waiting emergency calls. This included 6593 DCAs and 6763 solo responders. On 15 occasions, resuscitation was initiated by the attending ambulance resource. However, investigation of those incidents concluded that appropriate decision making had taken place and that no patient harm had resulted.

## Acknowledgements

We would like to thank Mark Faulkner for his contribution to historical data collection, and advanced paramedic practitioners in critical care for their daily contributions to the dataset.

## Author contributions

NB was responsible for data collection, and is the main author. CP is the secondary author. NB acts as the guarantor for this article.

## Conflict of interest

None declared.

## Ethics

Not required.

## Funding

None.
